# Bladder perforation due to laparoscopic peritoneal dialysis catheterization: A case report and literature review

**DOI:** 10.1097/MD.0000000000040444

**Published:** 2024-11-08

**Authors:** Xiaoyun Shao, Yanling Zhang, Weixing Xu

**Affiliations:** a Department of Intensive Care Unit, Shaoxing Second Hospital, Shaoxing, Zhejiang, China; b Department of Nephrology, Shaoxing Second Hospital, Shaoxing, Zhejiang, China; c Department of Gastrointestinal and Minimally Invasive Surgery, Shaoxing Second Hospital, Shaoxing, Zhejiang, China.

**Keywords:** bladder perforation, catheterization, laparoscopic, peritoneal dialysis

## Abstract

**Rationale:**

Complications related to the peritoneal dialysis (PD) catheter are the primary cause of treatment failure in PD, and bladder perforation is a rare complication of PD catheter placement. To date, there have been no reported cases of bladder perforation occurring during laparoscopic PD catheter placement.

**Patient concerns:**

An 80-year-old woman was admitted to Shaoxing Second Hospital due to a decade-long history of high blood creatinine levels. She was diagnosed with end-stage renal disease and underwent laparoscopic PD catheter placement. On the third day post-operation, she experienced frequent urination and urgency during her initial peritoneal dialysis fluid exchange.

**Diagnoses:**

The urine analysis indicated sterility. Both ultrasound and computed tomography scans suggested that the PD catheter was positioned in the bladder. We suspect a bladder perforation following laparoscopic placement of the PD catheter.

**Interventions:**

The patient underwent emergency surgery, during which the PD catheter was removed and subsequently replaced using laparoscopy. Additionally, due to a bladder perforation, peritoneal dialysis was temporarily suspended for 10 days postoperatively.

**Outcomes:**

On the 11th postoperative day, the patient underwent a low-dose peritoneal dialysis, and the procedure was uneventful.

**Lessons:**

During laparoscopy, PD catheter placement still carries the risk of rare complications such as bladder perforation. If postoperative PD catheter patients experience urinary urgency and frequency, there should be a high suspicion of bladder perforation. Early diagnosis and surgical intervention are crucial for improving patient prognosis.

## 1. Introduction

Peritoneal dialysis (PD), offering the benefits of stable hemodynamics, a low risk of bloodstream infection, preservation of residual renal function, and enhancement of quality of life,^[1]^ has emerged as the preferred modality for renal replacement therapy in patients with end-stage renal disease. Percutaneous puncture catheterization, surgical catheterization, and laparoscopic catheterization are the most common methods for PD catheterization. Advancements in PD catheterization technology have led to a gradual decrease in the incidence of complications related to catheterization. However, these complications continue to be a primary cause of treatment failure in PD. The main complications include infection-related issues (exit site and tunnel infections) and mechanical problems (catheter displacement or occlusion, dialysate leakage, hernia, internal organ injury, and bleeding).^[2]^ Hence, timely identification and correction of catheter-related complications are essential for enhancing the prognosis of PD patients.

Bladder perforation is a rare mechanical complication, most frequently observed in patients with diabetes, a history of abdominal surgery, intra-abdominal adhesions, and those who have not voided their bladder prior to surgery.^[3]^ After a patient undergoes PD catheterization and exhibits strong urgency to urinate, increased urine output, hematuria, marked bladder distension following infusion of peritoneal dialysis fluid, and signs of peritonitis, the possibility of bladder perforation should be considered.^[4]^

As far as we know, there have been only 15 reported cases of bladder perforation following PD catheter placement. This article presents a case of bladder perforation after laparoscopic PD catheter placement. Furthermore, we provide a comprehensive review of the techniques for catheter placement, clinical manifestations, therapeutic interventions, and outcomes associated with this rare complication documented in the existing literature.

## 2. Case presentation

On May 10, 2024, an 80-year-old female with a past medical history of symptomatic myomas who underwent hysterectomy, was admitted to the Nephrology Department of Shaoxing Second Hospital due to a decade-long elevation in serum creatinine levels. After admission to the hospital, the physical examination revealed both lower limbs edema, and the other vital signs were normal. Laboratory examination was as follows: blood urea nitrogen 21.9 mmol/L, serum creatinine 575 µmol/L, hemoglobin 12.6 g/dL. Computed tomography (CT) scan of the abdomen showed atrophy of both kidneys

The patient was diagnosed with end-stage renal disease and underwent PD regimen. We considered that this patient had a history of abdominal surgery, which may result in severe peritoneal tissue adhesion, complicating the percutaneous implantation of the PD catheter and leading to a poor prognosis. As a result, laparoscopic PD catheterization was recommended for this patient. The patient was unable to fully empty her bladder due to pre-procedural nervousness.

**Figure 1.**
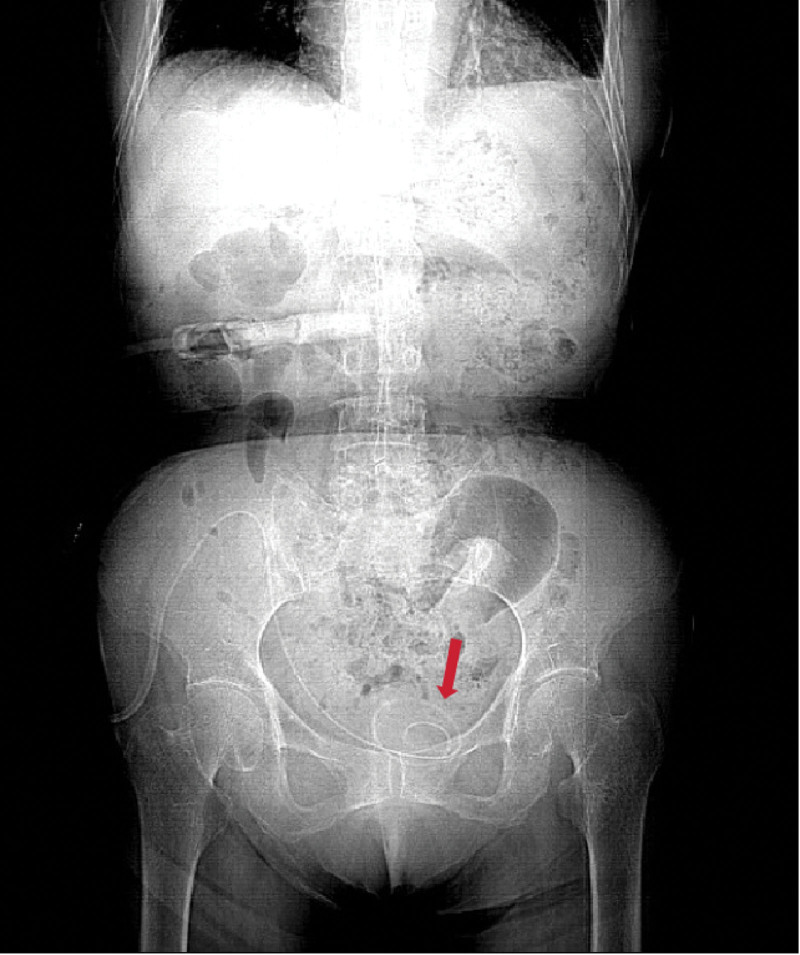
The X-ray indicates that the catheter is in the correct position.

**Figure 2.**
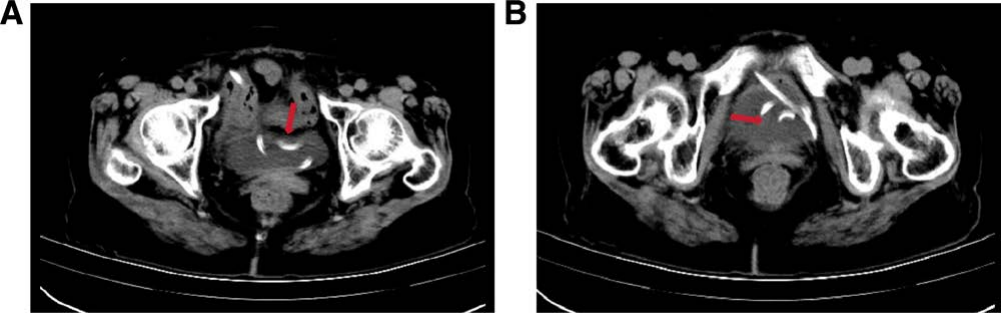
(A and B) The CT scan suggests that the PD catheter has penetrated the bladder. CT = computed tomography, PD = peritoneal dialysis.

The laparoscopic intra-operative findings were bladder overfilling and significant omental adhesions. Following the insertion of the PD catheter, drainage proceeded unobstructed. On post-surgery day 3, the patient experienced urgency and frequency of urination. Urinalysis revealed bland urine with a negative result for urinary tract infection. The X-ray indicates that the catheter is in the correct position (Fig. 1), while the CT scan suggests that the PD catheter has penetrated the bladder (Fig. 2).

**Figure 3.**
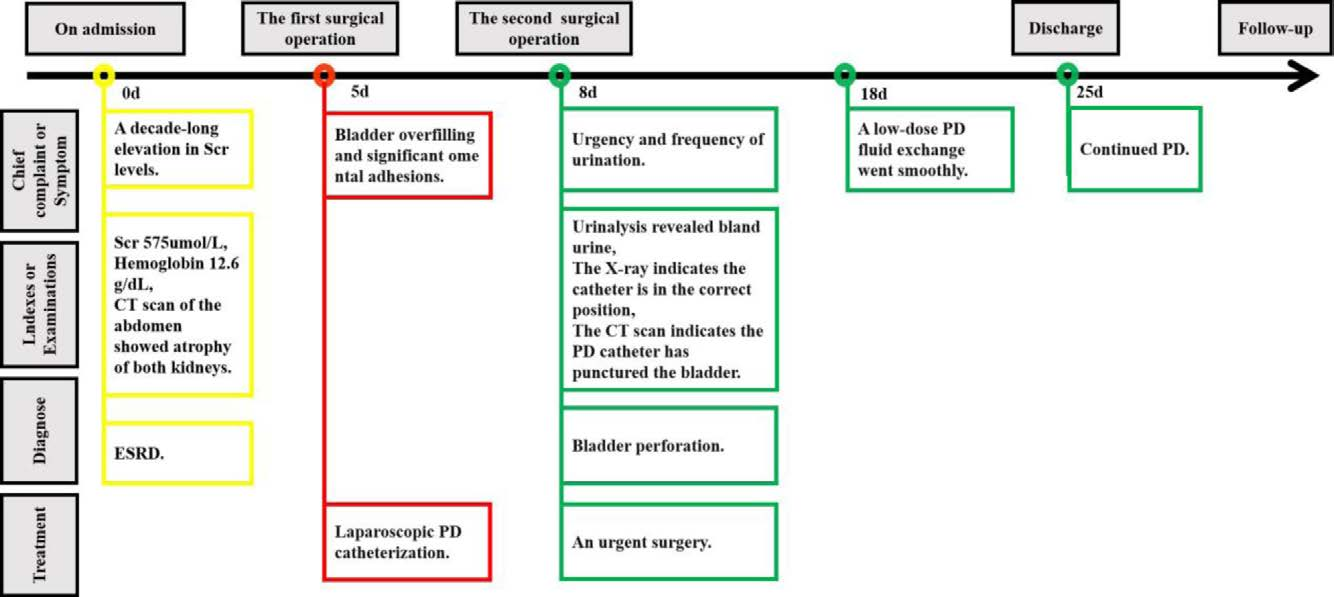
The entire process of diagnosis, treatment and outcome. ESRD = end-stage renal disease, PD = peritoneal dialysis.

We considered initiating an urgent surgery for a bladder perforation following laparoscopic PD catheterization. The PD catheter was removed and replaced with a new one in the right lower side. No evident active bleeding or fluid leakage was noted at the original peritoneal puncture site. The new catheter was positioned within the bladder-rectal depression and showed good flow when tested with saline. Then the incision was closed with sutures. For bladder injury, the urology consultation recommended a 10-day suspension of PD following surgery, with the possibility of conducting a cystography prior to resuming PD. By the 11th day post-2nd-surgery, the patient refused to undergo cystography and expressed a desire for a repeat low-dose PD fluid exchange. The subsequent dialysis procedure was conducted without any complications or urgent symptoms. Throughout the following week, the volume of dialysis fluid was gradually escalated while ensuring normal inflow and outflow rates. Based on these observations, we inferred spontaneous closure of the bladder perforation site. Figure 3 illustrates the timeline for diagnosis and treatment.

## 3. Discussion

PD is suitable for the clinical treatment of acute and chronic renal failure patients and has been widely used globally, becoming one of the important options for renal replacement therapy. Successful PD catheter placement is a prerequisite for the smooth conduct of PD. However, Bryk and Zhao^[5]^ and Pereira et al^[6]^ reported a failure rate of PD catheter placement of about 10% to 22%. Early complications such as surgical wound bleeding, catheter bleeding, organ perforation, and dialysate leakage through the wound necessitate surgical intervention; however, there remains a lack of systematic and comprehensive research. Bladder perforation is a very rare complication in PD catheter placement. All previous cases were about bladder perforation caused by percutaneous PD catheter placement. The reason may be poor surgical technique, lack of experience or inattention. When the initial symptom after surgery is urine leaking through the catheter, it indicates an emergency surgical indication exists. The 2023 SAGES guidelines indicate that laparoscopic PD catheter placement offers advantages such as shorter surgical duration, minimally invasive procedures, and reduced risk of complications^[7]^; however reports on bladder perforation resulting from laparoscopic PD catheter placement remain relatively scarce. This study presents the first case of bladder perforation following laparoscopic PD catheter placement in China.

We conducted a search of the PubMed database and excluded cases of bladder perforation resulting from trauma, as well as those with missing key data. A total of 15 cases of bladder perforation following PD catheter placement were included in the study, comprising 2 from Canada,^[8,9]^ 2 from the United States,^[10,11]^ 2 from China,^[12,13]^ 1 each from Saudi Arabia,^[14]^ Thailand,^[15]^ Poland,^[16]^ Turkey,^[17]^ the United Kingdom,^[18]^ India,^[19]^ Tunisia,^[3]^ Slovenia^[20]^ and Spain.^[21]^ Table 1 provides a summary of the clinical data related to bladder perforation subsequent to PD catheter placement.

**Table 1:** Case summary of bladder perforation due to laparoscopic PD catheterization.

References	Country	Gender	Age	Catheterization method	Catheterization time	Perforation symptoms	Infection	Outcome
Zhang et al (2023)^[13]^	China	Male	73	Unknown	1 year	Failed to drain	Peritonitis	PD catheter removed and shift to hemodialysis
El et al (2023)^[14]^	Saudi Arabia	Male	54	Laparoscopic-assisted approach	3 months	None	None	Renal transplantation
Jintanapramote et al (2023)^[15]^	Thailand	Male	93	Percutaneous Catheter Insertion	2 weeks	Cloudy PD effluent and urine volume increased	Peritonitis	PD catheter removed and shift to hemodialysis
Shi et al (2022)^[12]^	China	Male	64	Percutaneous Catheter Insertion	2 days	Urgent urination and gross hematuria	Urinary tract infection	PD catheter removed and shift to hemodialysis
Iwaniak et al (2022)^[16]^	Poland	Male	57	Percutaneous Catheter Insertion	1 day	Urine volume increased	None	Continued PD
Gulcan et al (2018)^[17]^	Turkey	Male	64	Percutaneous Catheter Insertion	2 days	Urgent urination	None	Continued PD
Riar et al (2018)^[9]^	Canada	Male	81	Percutaneous Catheter Insertion	1 week	Amber PD effluent	None	Continued PD
Yao et al (2018)^[8]^	Canada	Female	60	Laparoscopic-assisted approach	2.5 months	Urgent urination and urine volume increased	None	Continued PD
Elgaali et al (2017)^[18]^	UK	Male	60	Open surgical	2 weeks	Urine volume increased	None	Continued PD
Nasir et al (2013)^[19]^	India	Male	75	Percutaneous Catheter Insertion	1 week	Urgent urination	None	PD catheter removed and shift to hemodialysis
Ounissi et al (2012)^[3]^	Tunisia	Female	38	Unknown	3 years	Urgent urination and urine volume increased	None	PD catheter removed and shift to hemodialysis
Ekart et al (2006)^[20]^	Slovenia	Male	55	Percutaneous Catheter Insertion	1 day	Urgent urination	None	Continued PD
Moreiras et al (1997)^[21]^	Spain	Male	54	Unknown	2 weeks	Urgent urination	None	PD catheter removed and shift to hemodialysis
Rall et al (1993)^[11]^	United States	Male	59	Unknown	1 day	Failed to drain	None	PD catheter removed
Bamberger et al (1993)^[10]^	United States	Male	45	Open surgical	11 days	Failed to drain	None	Continued PD
Present report (2024)	China	Female	80	Laparoscopic-assisted approach	3 days	Urgent urination	None	Continued PD

PD = peritoneal dialysis.

Most of the patients with PD catheter-related bladder perforation were male (13/15), but there is no relevant study to demonstrate that gender is a factor affecting PD-related bladder perforation, which requires further research for confirmation. The most common clinical manifestations in these patients include urgency of urination (8/15) and increased urine output (5/15), and our patient also presented with frequency of urination, which resembles the symptoms of a urinary tract infection. Other clinical manifestations encompass drainage obstruction, cloudy dialysate, hematuria, etc, all associated with peritoneal dialysis or bladder. In a case reported by El et al,^[14]^ the PD catheter penetrated the entire bladder without any clinical manifestations and was only discovered during kidney transplantation. We deduced that although this patient’s catheter had penetrated the bladder, it did not retract into it; thus, the bladder was still considered a closed container. Consequently, diagnosing or identifying bladder perforation based solely on clinical manifestations can be challenging.

The PD catheter implantation techniques mainly include surgical incision, laparoscopy, and percutaneous puncture.^[22]^ Among these, surgical incision and laparoscopy can be classified as visual insertion techniques, while percutaneous puncture can be considered a blind insertion technique. The predominant placement methods for PD catheters in Table 1 are percutaneous puncture (7/15), laparoscopy (2/15), and open surgery (2/15). Three cases did not specify the placement methods. A meta-analysis incorporating 10 clinical studies indicates that the incidence of internal organ perforation caused by various placement methods, including surgical incision, laparoscopy, and percutaneous puncture, is <1%.^[23]^ Furthermore, the study findings demonstrate that the incidence of internal organ perforation using blind percutaneous insertion of PD catheters is higher than that associated with direct visual insertion techniques.^[24]^ Therefore, it is recommended to employ direct visual insertion techniques such as laparoscopy or open insertion surgery for patients with specific medical histories or anatomical abnormalities. In this case, the patient had laparoscopic surgery, but unfortunately experienced a bladder perforation. Subsequently, the surgeon performed a postoperative trace analysis, revealing several contributing factors to the accidental catheter puncture into the bladder: Firstly, the patient had a history of pelvic surgery resulting in extensive adhesions of the small intestine in the lower abdominal wall, leading to fixation of the bladder at a higher position below the incision; secondly, preoperatively, the patient did not void his bladder, leaving it distended. Additionally, there was a deviation from previous procedures as the surgeon stood on the patient’s right side and inserted the catheter on the outer aspect of rectus abdominis muscle on that side. This necessitated significant force to control and advance the inner core guidewire through peritoneum. The above 3 factors combined to cause the accidental puncture of the catheter into the bladder.

Most bladder perforations occurred in the perioperative period (11/15), ranging from 1 day to 2 weeks postoperatively. Nearly all patients with perforations exhibited clinical symptoms, such as urgency, cloudy dialysate, and failure to drain. Only El et al^[14]^ reported a case in which the patient was asymptomatic because the PD catheter traversed the bladder and reentered the abdominal cavity without affecting dialysate flow. Complications associated with bladder perforation may include sepsis, peritonitis, abscesses, urinary fistulas, cysts, and electrolyte imbalances.^[4]^ In this study, the primary complications observed were peritonitis and urinary tract infections; however, the majority of patients did not experience any complications. This observation may be attributed to the penetrating nature of bladder injuries in this study. As for the outcomes of bladder perforation, nearly half of the patients (7/15) continued PD after the self-repair of the bladder, while the other half were switched to HD.

Currently, X-ray or CT cystography is commonly utilized in clinical practice for the investigation and assessment of bladder injury. However, clinicians more frequently opt for CT cystography due to its superior specificity, sensitivity, and other advantages.^[25]^ In this instance, we opted not to conduct a CT urography for further clarification, but instead scheduled a urological CT scan due to our patient’s status as part of the end-stage renal disease population. The administration of contrast agent would impose additional strain on the kidneys, potentially precipitating an acute need for emergency dialysis. Fortunately, our patient maintained peritoneal dialysis (PD) following emergency surgery for a bladder perforation, without experiencing any additional complications during the perforation procedure.

## 4. Conclusion

In conclusion, iatrogenic bladder injury is relatively common in urological surgery, while bladder perforation caused by PD catheters is rare. However, with early diagnosis and prompt treatment, patients generally have a favorable prognosis. Additionally, during the postoperative evaluation phase, we considered that the contrast agent used in emission computed tomography bypasses renal metabolism. In future similar cases, direct emission computed tomography examination through the PD catheter can promptly assess the extent of bladder injury.

## Author contributions

**Conceptualization:** Xiaoyun Shao.

**Data curation:** Xiaoyun Shao.

**Investigation:** Xiaoyun Shao, Yanling Zhang.

**Methodology:** Xiaoyun Shao.

**Project administration:** Xiaoyun Shao.

**Supervision:** Xiaoyun Shao, Yanling Zhang, Weixing Xu.

**Validation:** Xiaoyun Shao, Yanling Zhang, Weixing Xu.

**Writing – original draft:** Xiaoyun Shao, Yanling Zhang.

**Writing – review & editing:** Xiaoyun Shao, Weixing Xu.
